# Thickness-Prediction Method Involving Tow Redistribution for the Dome of Composite Hydrogen Storage Vessels

**DOI:** 10.3390/polym14050902

**Published:** 2022-02-24

**Authors:** Hui Wang, Shuang Fu, Yizhe Chen, Lin Hua

**Affiliations:** 1 Hubei Key Laboratory of Advanced Technology for Automotive Components, Wuhan University of Technology, Wuhan 430070, China; huiwang@whut.edu.cn (H.W.); fushuang0529@whut.edu.cn (S.F.); 2 Hubei Engineering Research Center for Green & Precision Material Forming, Wuhan 430070, China; linhua@whut.edu.cn; 3 Hubei Collaborative Innovation Center for Automotive Components Technology, Wuhan 430070, China

**Keywords:** composite hydrogen storage vessels, fiber slippage, tow redistribution, dome thickness, prediction method, progressive damage, load-bearing capacity

## Abstract

Traditional thickness-prediction methods underestimate the actual dome thickness at polar openings, leading to the inaccurate prediction of the load-bearing capacity of composite hydrogen storage vessels. A method of thickness prediction for the dome section of composite hydrogen storage vessels was proposed, which involved fiber slippage and tow redistribution. This method considered the blocking effect of the port on sliding fiber tows and introduced the thickness correlation to predict the dome thickness at polar openings. The arc length corresponding to the parallel circle radius was calculated, and then, the actual radius values corresponding to the bandwidth were obtained by the interpolation method. The predicted thickness values were compared with the actual measured thickness. The maximum relative error of the predicted thickness was 4.19%, and the mean absolute percentage error was 2.04%. The results show that the present method had a higher prediction accuracy. Eventually, this prediction method was used to perform progressive damage analysis on vessels. By comparing with the results of the cubic spline function method, the analysis results of the present method approached the actual case. This showed that the present method improved the accuracy of the design.

## 1. Introduction

As pollution-free energy, hydrogen energy has become a new energy to replace traditional fossil energy. The burning of conventional fossil fuels produces greenhouse gas and harmful gases, while hydrogen only produces water [1]. Composite hydrogen storage vessels, as the primary equipment of high-pressure hydrogen storage, have the advantages of lightweight and reliable performance [2]. The wide application of hydrogen energy is inseparable from hydrogen storage vessels.

The filament-winding process has been extensively researched and applied [3,4]. The filament-winding process is usually used in the manufacture of hydrogen storage vessels. The geometry and mechanical properties analysis of hydrogen storage vessels, especially in the dome section, are very complicated because of the characteristics of the process and materials. In addition, the thickness and winding angle of composite layers at the dome section of vessels will change significantly, which affects the mechanical properties of composite layers [5,6].

Nowadays, finite-element analysis technology plays an increasingly important role in designing high-performance composite hydrogen storage vessels [7]. The more accurate the hydrogen storage vessel model established, the more accurate the results of the finite-element analysis will be [8]. The changes in angle and thickness at the dome section need to be considered in the accurate performance prediction of a hydrogen storage vessel. It is difficult to predict the dome thickness of vessels because of fiber slippage during the winding process. Therefore, accurate thickness prediction is a prerequisite for the performance prediction of hydrogen storage vessels. The design reliability of vessels can be increased by accurate thickness prediction and finite-element analysis, achieving more effective optimization schemes. The dome thickness of hydrogen storage vessels was predicted to establish the finite-element model to research the performance of the composite hydrogen storage vessel by researchers [9].

However, traditional thickness-prediction methods were only accurate outside the two bandwidths of polar openings. The influence of fiber width was not considered, which caused the predicted thickness value at polar openings to be infinite and did not match actual measurement thickness [10]. Wang et al. [11,12] proposed a thickness-prediction method, which used a cubic spline function and assumed that the amounts of the composite material were constant within the two bandwidths of polar openings. The method provided more accurate thickness-prediction results, but it did not consider the slippage effect of fiber tows during the winding process, which made prediction results at polar openings far lower than actual measurement thickness.

In recent years, many researchers also proposed new thickness-prediction methods. Leh et al. [13] combined existing winding-angle and thickness-prediction methods [8,11,12] to propose a model for predicting the dome geometric characteristics of multi-layer composite pressure vessels, but the problems existing in the existing methods was not improved. Zu et al. [14,15] used a three-dimensional laser scanner to measure the vessel’s profile after winding and obtained the thickness distribution of the dome section. However, the method needed thickness values provided by experiments, which limited the application of this method. Che et al. [16] proposed a prediction method for the thickness of the dome section. This method took the effect of fiber overhead caused by hoop winding, but this method did not consider the influence of bandwidth on the fiber thickness at polar openings. Akhtar et al. [17] presented the analytical modeling involving kinematic constraints based on a geodesic trajectory to predict thickness change of dome section. However, the method did not consider the slippage effect of fiber tows during the winding process, and it needed to divide the dome section into multiple zones for calculation. The number of divided zones affected prediction accuracy.

The predicted thickness values could provide a basis for the establishment of a finite-element model (FEM) of composite hydrogen storage vessels. A progressive damage model has been widely used in progressive damage and failure analysis of composite masteries [18]. Composite material progressive damage analysis method could simulate complex stress conditions and material damage state of composite vessels under internal pressure. Burst pressure and burst mode of composite hydrogen storage vessels could be better predicted by progressive damage analysis [19,20].

The progressive damage analysis of composite hydrogen storage vessels included the establishment of FEM and the analysis of results. ANSYS ACP was used by Kangal et al. [21] to analyze the progressive damage of the glass fiber/carbon fiber hybrid composite pressure vessel. The results of hydrostatic pressure tests were compared with the results of finite-element simulation analysis and showed that numerical simulation results were in good agreement with experimental results. However, the thickness and angle variations were obtained from CADWIND, and the simulation results of dome thickness obtained from CADWIND were inaccurate. A multiscale progressive damage analysis method was proposed by Lin et al. [22], which introduced representative volume elements (RVEs). Puck failure criterion was adopted to predict the burst pressure of composite pressure vessels, but the vessel was modeled by the plug-in WCM. There were some differences between the actual shell composite dimensions and those modeled by WCM. A progressive damage method was used by Alam et al. [23] to investigate the influence of winding angle, the number of layers, and the sequence of layers on the burst strength of composite pressure vessels. However, the method only compared the burst pressure values corresponding to different winding angles, numbers of layers, and sequences of layers without further research on its causes. ANSYS ACP software was used by Sharma et al. [24] to perform progressive failure analysis of composite hydrogen storage vessels. The influence of the interlayer hybridization effect on the burst pressure of composite vessels was analyzed. However, the dome section was divided into multiple areas in that model, resulting in a discontinuous angle and thickness of the dome section, which was different from the actual angle and thickness. A composite material progressive damage model was established by Hu et al. [25] using ABAQUS software. The Hashin failure criterion was used to predict the burst pressure of composite hydrogen storage vessels with different layer-up sequences. The liner used in the article was different from the conventional vessel liner, and the conclusion may not apply to others.

Progressive damage analysis could better simulate the complex stress condition and material damage state of composites [26]. However, previous literature [27,28] mostly used WCM or CADWIND software to model the composite hydrogen storage vessels, and the thickness values at the dome section could not be predicted well by the software, thus affecting the accuracy of the FEM. To accurately predict the burst pressure of composite hydrogen storage vessels, it is beneficial to study a more accurate prediction method of dome thickness.

In this paper, a method of thickness prediction for the dome section of composite hydrogen storage vessels was proposed, which involved the effect of fiber slippage and the redistribution of fiber tows. This method solved the problem that the traditional cubic spline function method underestimated the dome thickness at polar openings. On this basis, this prediction method was used to perform progressive damage analysis on composite hydrogen storage vessels. The influence of the reaming process and the layer-up sequence was studied on load-bearing capacity and damage evolution of hydrogen storage vessels. The analysis results of the present method were compared with the results of the cubic spline function method.

## 2. Analytical Model for Dome Thickness Prediction

An accurate dome-thickness-prediction model was a key point in the design process of composite hydrogen storage vessels. The results accuracy of finite-element analysis was affected by the prediction accuracy. The cubic spline function prediction method proposed by Wang et al. [12] was a relatively accurate prediction method. However, the thickness-prediction values of the cubic spline function method at polar openings were much lower than the actual measurement. The effect of fiber slippage on dome thickness at polar openings was not considered. The cubic spline function may become undefined when using a large initial winding angle and large bandwidth. A new prediction method is proposed in the following text.

### 2.1. Dome Thickness at Polar Openings

**Figure 1:**
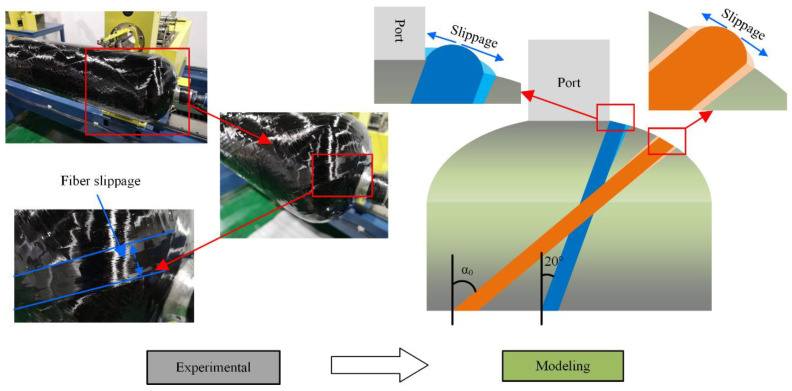
Schematic diagram of fiber slippage at different winding angles.

Figure 1 shows a schematic diagram of fiber slippage during the winding process. Two situations need to be considered when predicting the dome thickness at polar openings: (1) dome thickness at polar openings before reaming; and (2) dome thickness at polar openings after reaming. The dome thickness at polar openings should be calculated separately based on the above two cases.

#### 2.1.1. Dome Thickness before Reaming

The wet winding process was usually used to manufacture hydrogen storage vessels. The fiber tows slid to both sides under the action of winding tension and resin infiltration when fiber tows were wound around polar openings. The redistribution of fiber tows was caused by the effect of fiber slippage. However, the fiber tows cannot slide further because of the blocking effect of the port, which leads to the fiber stacking around polar openings. The thickness of polar openings cannot be calculated according to the theory of the same amounts of fiber tows. The thickness at polar openings and the thickness one bandwidth away from polar openings were affected by factors such as the slipping, realignment, and roving separation of fiber tows. There was a correlation between the thickness of the two places, considering the redistribution of the fiber tows near polar openings. The formula for calculating the dome thickness at polar openings before reaming was expressed as follows:

(1) $$t\left(r\right)=\frac{m_{R}\times n_{R}}{\pi }\times \arccos\left(\frac{r_{0}}{r_{b}}\right)\times t_{p}\times \gamma$$

where $m_{R}$ stands for the number of fiber tows at the cylindrical section, $n_{R}$ stands for the number of layers in the cylindrical section, $r_{0}$ is the radius of the polar opening, r is the radius of the parallel circle, $t_{p}$ is the thickness of a fiber tow, b is the width of a fiber tow, γ is the thickness correlation coefficient, and the value is 0.2~0.3.

#### 2.1.2. Dome Thickness after Reaming

Fiber stacking occurred near polar openings if helical layers were wound at a single helical angle. Winding difficulties and stress concentration in composite layers were caused by fiber stacking, which affected the load-bearing capacity of the vessels. Therefore, the reaming process was used to reduce thickness accumulation and fiber stacking at the dome section. The observation of the fiber slippage effect through actual winding experiments is shown in Figure 1. The fiber tows during the winding process after the reaming process had no longer been obstructed by the port. However, the resin-impregnated fiber tows still slid to both sides under the action of winding tension. As a result, the thickness of the fiber tows around actual polar openings decreased, and the width of fiber tows increased. From the winding measurement, the bandwidth used in the calculation in this paper was 1.2 times the actual bandwidth, and the thickness was divided by 1.2.

### 2.2. Revised Model of Radius Corresponding to Bandwidth

**Figure 2:**
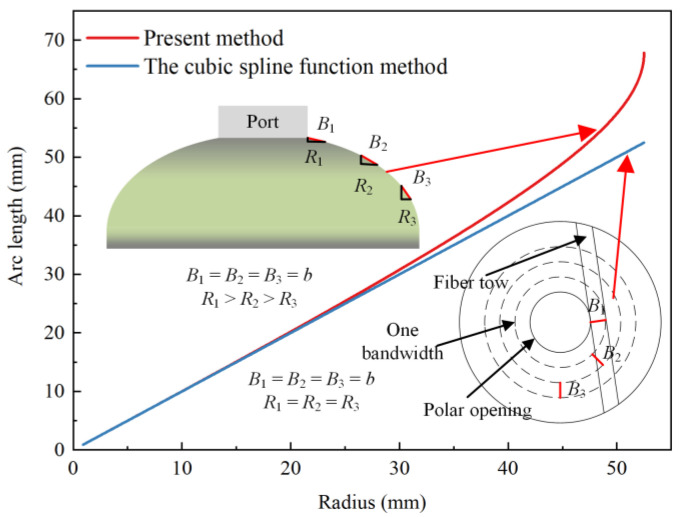
Comparison of analytical results of the arc length corresponding to the radius of parallel circle between two methods.

The radius of the cubic spline function method was calculated by projecting fiber tows trajectory onto the horizontal plane. The formula ($r_{b}=r_{0}+b,r_{2b}=r_{0}+2b$) was workable when polar openings radius and bandwidth were small. However, the radius of polar openings of composite layers increased after the reaming process. The projected length of fiber tows bandwidth on the horizontal plane decreased, as shown in Figure 2. The traditional cubic spline function was not suitable to predict the thickness of the dome section. The actual radius values of $r_{b}$, $r_{2b}$ corresponding to the bandwidth were calculated by the actual arc length in this paper. MATLAB R2020b [29] software calculated the arc length corresponding to parallel circle radius. The values used by the cubic spline function prediction method and the present method are shown in Figure 2. Thereafter, the values of $r_{b}$, $r_{2b}$ corresponding to bandwidth were calculated by the interpolation method.

### 2.3. Revised Model of Fiber Volume within Two Bandwidths

When using the cubic spline function prediction method, the thickness was set as a fixed value during the calculation process for amounts of composite material within two bandwidths of polar openings. In the present method, the values of dome thickness were variable, which varied with the radius of the parallel circle. The amounts of fiber tows within two bandwidths could be expressed as follows:

(2) $$\begin{array}{ll} V_{const}= & \int_{r_{0}}^{r_{b}}2\pi r\times \frac{m_{R}\times n_{R}}{\pi }\times \arccos\left(\frac{r_{0}}{r}\right)\times t_{p}dr+\;\;\int_{r_{b}}^{r_{2b}}2\pi r\times \\ & \frac{m_{R}\times n_{R}}{\pi }\times \left[\arccos\left(\frac{r_{0}}{r}\right)-\arccos\left(\frac{r_{0}+b}{r}\right)\right]\times t_{p}dr \end{array}$$

The analytical model for dome thickness prediction could be expressed as follows:

(3) $$t\left(r_{i}\right)=m_{1}\times r_{i}^{0}+m_{2}\times r_{i}^{1}+m_{3}\times r_{i}^{2}+m_{4}\times r_{i}^{3}\left(r_{0}\leq r\leq R\right)$$

The unknown coefficients $m_{1}$, $m_{2}$, $m_{3}$, and $m_{4}$ could be solved from:

(4) $$\begin{array}{cc} \left[\begin{array}{c} m_{1}\\ m_{2}\\ m_{3}\\ m_{4} \end{array}\right]= & {\left[\begin{array}{cccc} 1 & r_{0} & r_{0}^{2} & r_{0}^{3}\\ 1 & r_{0} & r_{2b}^{2} & r_{2b}^{3}\\ 0 & 1 & 2r_{2b} & 3r_{2b}^{2}\\ \pi \left(r_{2b}^{2}-r_{0}^{2}\right) & \frac{2\pi }{3}\left(r_{2b}^{3}-r_{0}^{3}\right) & \left(r_{2b}^{4}-r_{0}^{4}\right) & \left(r_{2b}^{5}-r_{0}^{5}\right) \end{array}\right]}^{-1}\times \\ & \left[\begin{array}{c} \frac{m_{R}\times n_{R}}{\pi }\times \arccos\left(\frac{r_{0}}{r_{b}}\right)\times t_{p}\times \gamma \\ \frac{m_{R}\times n_{R}}{\pi }\times \left[\arccos\left(\frac{r_{0}}{r_{2b}}\right)-\arccos\left(\frac{r_{b}}{r_{2b}}\right)\right]\times t_{p}\\ \frac{m_{R}\times n_{R}}{\pi }\times \left(\frac{r_{0}}{r_{2b}\times \sqrt{r_{2b}^{2}-}r_{0}^{2}}-\frac{r_{b}}{r_{2b}\times \sqrt{r_{2b}^{2}-}r_{b}^{2}}\right)\times t_{p}\\ V_{const} \end{array}\right] \end{array}$$

where *V_const_* could be calculated by:

(5) $$\begin{array}{cc} V_{const}= & \int_{r_{0}}^{r_{b}}2\pi r\times \frac{m_{R}\times n_{R}}{\pi }\times \arccos\left(\frac{r_{0}}{r}\right)\times t_{p}dr+\int_{r_{b}}^{r_{2b}}2\pi r\times \\ & \frac{m_{R}\times n_{R}}{\pi }\times \left[\arccos\left(\frac{r_{0}}{r}\right)-\arccos\left(\frac{r_{0}+b}{r}\right)\right]\times t_{p}dr \end{array}$$

### 2.4. Verification of the Analytical Model

**Figure 3:**
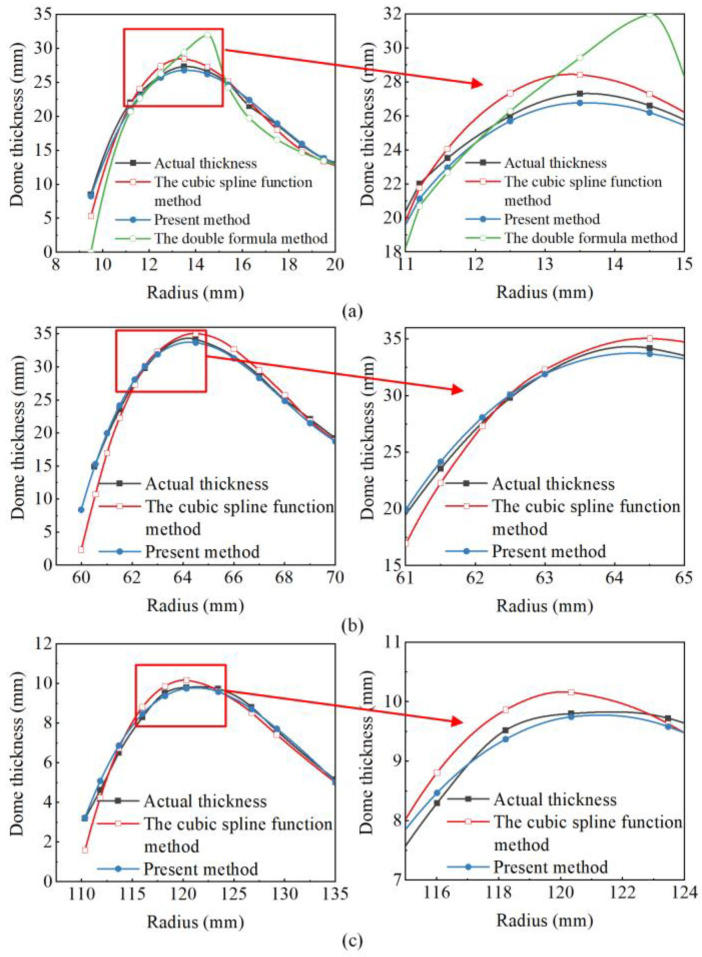
Comparison of analytical and measurement results for dome thickness distribution within two bandwidths: (**a**) Type A vessel, (**b**) Type B vessel, and (**c**) Type C vessel.

The profiles of three composite vessels in published literature [11,12] were selected to validate the accuracy of the analytical model for dome thickness prediction. The geometric parameters of composite vessels are shown in Table 1. The prediction results are shown in Figure 3. The actual measured thickness values were obtained from the literature [11,12]. The present method had a better agreement with the actual measured thickness values at polar openings and within two bandwidths than the cubic spline function method. Table 2 shows the relative error between predicted values and actual values of type A vessel within two bandwidths.

The double formula method [30] provided a reference for these models. It can be seen in Table 2 that the maximum relative error of the double formula method was 20.23%, the mean absolute percentage error was 6.66%, and the root-mean-square error was 1.95 mm. The maximum relative error of the cubic spline function method was 5.15%, the mean absolute percentage error was 3.22%, and the root-mean-square error was 0.70 mm. The maximum relative error of the present method was 4.19%, the mean absolute percentage error was 2.04%, and the root-mean-square error was 0.48 mm. Compared with the cubic spline function method, the maximum relative error of the present method was reduced by 18.6%, the mean absolute percentage error by 36.6%, and the root-mean-square error by 31.4%. The present method predicted the thickness distribution of the dome section more accurately.

## 3. Numerical Modeling

### 3.1. Winding Angle and Thickness

The winding angle and thickness were needed in the finite-element modeling of composite hydrogen storage vessels. The winding angle along the cylindrical section directly followed the design’s winding angle. The winding angle of the helical layers at the dome section changed from the initial winding angle (*α*_0_) to 90°. Therefore, the angle of each point of the dome section along the meridian was different. The geodesic trajectories were used for the winding process because of the use of equal domes. The helical winding angle over the dome section was generally assumed to follow geodesic formula [31]:

(6) $$a=\arcsin\left(r_{0}/r\right)$$

**Figure 4:**
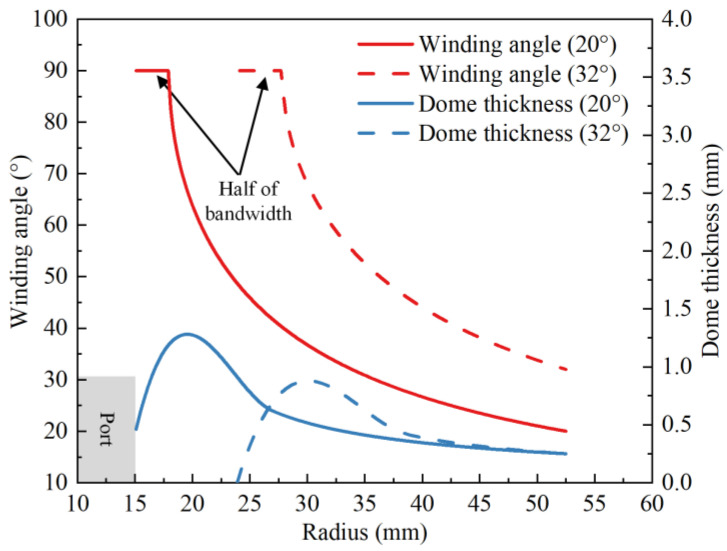
Analytical results of the variations of winding angle and dome thickness.

The values of dome thickness were obtained with the analytical model from Section 2. The winding angle and dome thickness distribution at the dome of the vessel are shown in Figure 4.

The helical winding angle and the minimum burst pressure of vessels were needed when calculating the thickness of the composite layers of the cylindrical section. The safety factor was set as 2.25 for the 70 MPa hydrogen storage vessel in this paper. Therefore, the minimum burst pressure should not be less than 2.25 times the working pressure.

The geodesic winding method was adopted since a pressure vessel with equal domes was used. The minimum angle of helical winding was calculated by the geodesic formula. The netting theory was commonly used to describe the minimum thickness *t_αk_* and *t_θk_* of helical and hoop layers, and its expression was:

(7) $$\{\begin{array}{l} t_{\alpha k}=\frac{Rp_{b}}{2k_{s}\sigma_{fb}\cos^{2}\alpha }\\ t_{\theta k}=\frac{Rp_{b}}{2\sigma_{fb}}\left(2-\frac{1}{k_{s}}\tan^{2}\alpha \right) \end{array}$$

(8) $$t_{k}=t_{\alpha k}+t_{\theta k}=\frac{\left(2k_{s}+1\right)Rp_{b}}{2k_{s}\sigma_{fb}}$$

where *t_αk_* and *t_θk_* are the thicknesses of helical layers and hoop layers, *t_k_* is the total thickness of composite layers at the cylindrical section, *α* is the helical winding angle, *R* is the radius of the cylindrical section, and *p_b_* is the minimum burst pressure.

The strength at the dome section was only determined by helical layers. The stress equilibrium factor *k_s_* needed to be introduced to increase the thickness of helical layers to strengthen the dome section. The stress equilibrium factor ranged from 0.6 to 0.8 to ensure that the burst point was at the cylindrical section.

The thickness of helical layers increased when the stress equilibrium factor was smaller. The burst point occurred in the cylindrical section of vessels. The burst mode of vessels was safer, but the thickness of composite layers increased. The value of the stress equilibrium factor was 0.7 in this paper. The initial winding angle *α* = 20°, the fiber tape width *b* = 6 mm, and the fiber tape thickness *t_p_* = 0.125 mm. A total of 26 hoop layers and 24 helical layers were wound.

### 3.2. Layer-Up Design Schemes

**Figure 5:**
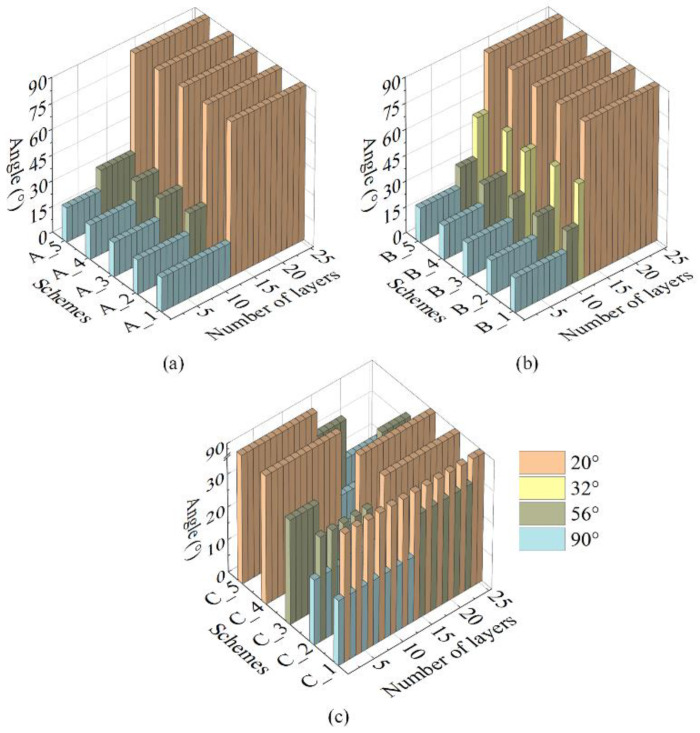
Layer-up design schemes used in the finite-element modeling: (**a**) Group A, (**b**) Group B, and (**c**) Group C.

Different layer-up design schemes were used, as shown in Figure 5, to study the influence of the reaming process and layer-up sequence on the load-bearing capacity of vessels. The schemes included different layer-up sequences and helical winding angles. The layer-up schemes were divided into three groups A, B, and C, and each group included five schemes. The thickness of helical layers and hoop layers in these schemes was calculated by the netting theory. All layer-up design schemes had the same number of layers and thicknesses. The scheme A_1 was the initial design scheme. The schemes of group A used single reaming and increased reaming layers. The schemes of group B used double reaming and increased reaming layers. The schemes of group C changed the layer-up sequence of helical layers and hoop layers based on scheme A_4. Each column in Figure 5 represents a complete hoop layer (±90°) or helical layer (±*α*). The horizontal axis represents the number of layers from inside to outside, and the vertical axis represents the winding angle.

### 3.3. Finite-Element Model

**Figure 6:**
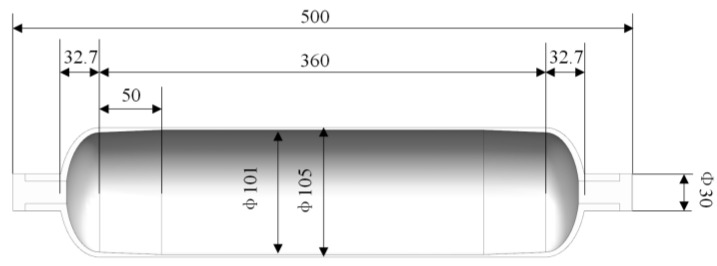
Cross-section of lining structure.

The FEM of the composite hydrogen storage vessel in this paper was a 70 MPa type III vessel. The liner was made of aluminum alloy 6061-T6, as shown in Figure 6, to meet the requirements of lightweight and hydrogen corrosion resistance of the hydrogen storage vessel. The length of the aluminum liner was 500 mm, the outer diameter was 105 mm, the inner diameter was 101 mm, and the dome section height was 32.7 mm.

**Figure 7:**
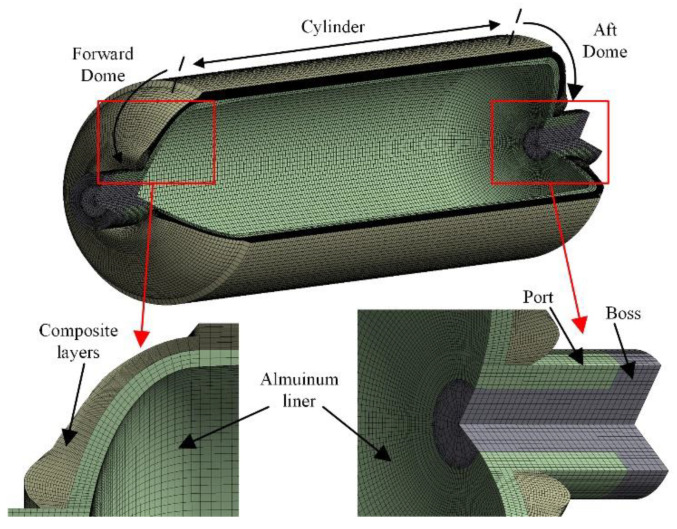
Finite-element model of the composite hydrogen storage vessel.

As shown in Figure 7, a finite-element model of the type III storage vessel was established to predict the burst pressures of composite hydrogen storage vessels. The cyclic symmetry was applied to improve computer efficiency, and a twelfth model was used to analyze vessels. The internal pressure was applied to the internal surface of the aluminum liner. The axial displacement of the vessel was restricted. Aluminum alloy 6061-T6 was used as the liner of vessels. The liner was modeled using a twenty-node cubic solid element (SOLID186) considering the nonlinearity of aluminum alloy material. The T700 fiber-reinforced composite was used as the composite layers of vessels. The composite layers were modeled using an eight-node structural solid shell element (SOLSH190). The aluminum liner and the composite layers were bonded together.

**Figure 8:**
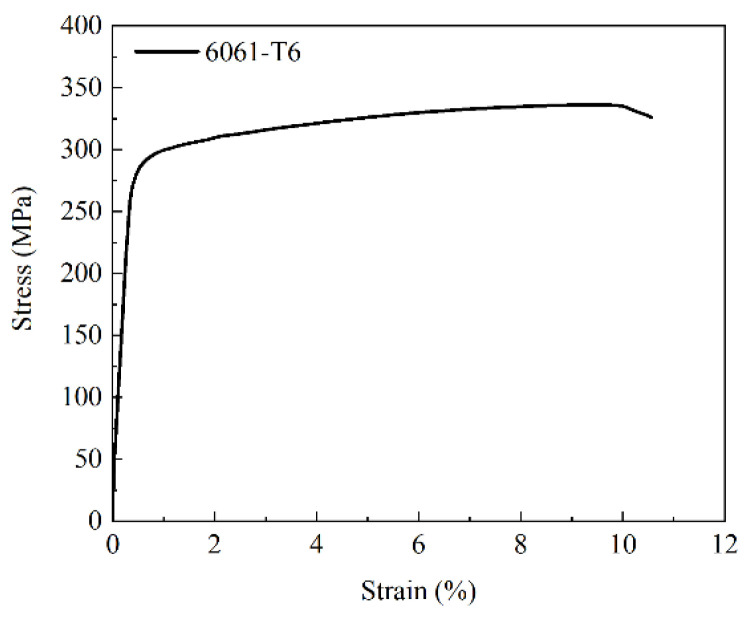
Stress-strain curve of the aluminum alloy.

The liner of the vessel was made of aluminum alloy (6061-T6), and the material model was isotropic and elastoplastic. The bilinear isotropic hardening model was used for the plastic region. The material parameters and stress-strain curve of the aluminum liner obtained from the literature [14] are shown in Figure 8 and Table 3. The composite layers of the vessel were made of T700 fiber-reinforced composites, and the material model was transversely isotropic. The material parameters of the T700 fiber-reinforced composites obtained from the literature [14] are listed in Table 4 and Table 5.

### 3.4. Progressive Damage Model

**Figure 9:**
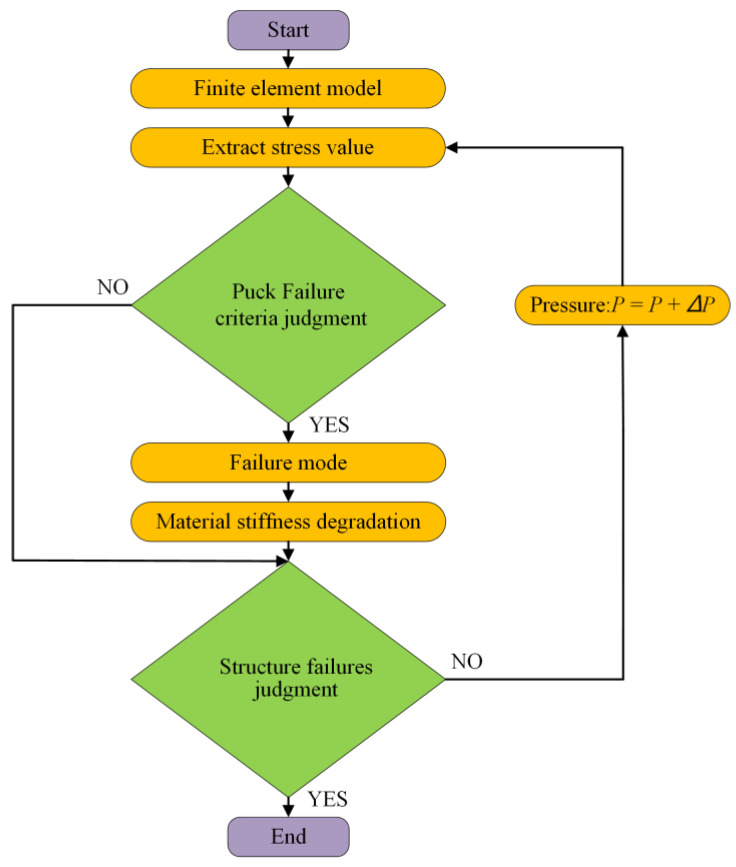
Flow chart of progressive damage process.

A progressive damage model was used to predict structural damage of composite layers and the burst pressure of vessels. The model could accurately predict mechanical responses and the ultimate load-bearing capacity of composite materials. A damage initiation criterion was defined to determine the failure of composite material. The damage evolution law controlled the stiffness reduction of the materials. This progressive damage analysis process is shown in Figure 9. First, a finite-element model of the composite hydrogen storage vessel was established. The internal pressure of the vessel was loaded, and stress calculation was performed. Puck failure criterion was used. A material-stiffness degradation model was adopted according to the corresponding failure mode after the material was damaged. Finally, the calculation was terminated when the hydrogen storage vessel was structurally damaged.

The Puck failure criteria [32] were employed to predict the damage onset of the composite layers. The failure model of composite layers was generally classified as fiber tension and compressive breakage, matrix tensile, and compressive failure.

The Puck failure criterion included fiber failure (*FF*) and inter-fiber failure (*IFF*). The fiber failure criterion was written as follows:

(9) $$\begin{array}{l} f_{E,FF}=\frac{1}{\pm R_{∥}^{t,c}}\left[\sigma_{1}-\left(\nu_{⊥∥}-\nu_{⊥∥f}\times m_{\sigma f}\frac{E_{∥}}{E_{∥f}}\right)\left(\sigma_{2}+\sigma_{3}\right)\right]\\ with\;\;\;\;\;\;\;\;\;\;\;\;\;\;\;\;\;R_{∥}^{t}\;\;\;\;\;for\;\left[\ldots \right]\geq 0\\ \;\;\;\;\;\;\;\;\;\;\;\;\;\;\;\;\;\;\;\;-R_{∥}^{c}\;\;\;\;\;for\;\left[\ldots \right]\leq 0 \end{array}$$

where $f_{E,FF}$ is the fiber failure stress exposure of the lamina; $R_{∥}^{t}$ is the tensile strengths of the materials; $R_{∥}^{c}$ is the compressive strengths of the materials; $\sigma_{1}$, $\sigma_{2}$ and $\sigma_{3}$ are the normal stresses in a unidirectional layer; $E_{∥}$, $E_{∥f}$ are the modules of the composite material and fiber direction; $\nu_{⊥∥}$, $\nu_{⊥∥f}$ are the major Poisson’s ratios of the lamina and fibers; and $m_{\sigma f}$ is the stress magnification factor for transverse stresses in the fibers and was set as 1.1.

The formulations for the stresses $\sigma_{n}$, $\tau_{nt}$ and $\tau_{n1}$ in an arbitrary plane with the inclination angle *θ* were:

(10) $$\begin{array}{l} \sigma_{n}\left(\theta \right)=\sigma_{2}\times \cos^{2}\theta +\sigma_{3}\times \sin^{2}\theta +2\times \tau_{23}\times \sin\theta \times \cos\theta \\ \tau_{nt}\left(\theta \right)=\left(\sigma_{3}-\sigma_{2}\right)\times \sin\theta \times \cos\theta +\tau_{23}\times \left(\cos^{2}\theta -\sin^{2}\theta \right)\\ \tau_{n1}\left(\theta \right)=\tau_{31}\times \sin\theta +\tau_{21}\times \cos\theta \end{array}$$

The Puck IFF criterion was written as follows:

For *σ_n_*(*θ*) ≥ 0:

(11) $$f_{E,IFF}\left(\theta \right)=\sqrt{\left[{\left(\frac{1}{R_{⊥}^{At}}-\frac{p_{⊥\psi }^{t}}{R_{⊥\psi }^{A}}\right)}^{2}\sigma_{n}\left(\theta \right)\right]+{\left(\frac{\tau_{nt}\left(\theta \right)}{R_{⊥⊥}^{A}}\right)}^{2}+{\left(\frac{\tau_{n1}\left(\theta \right)}{R_{⊥∥}}\right)}^{2}}\;\;\;+\frac{p_{⊥\psi }^{t}}{R_{⊥\psi }^{A}}\sigma_{n}\left(\theta \right)$$

For *σ_n_*(*θ*) < 0:

(12) $$f_{E,IFF}\left(\theta \right)=\sqrt{{\left(\frac{\tau_{nt}\left(\theta \right)}{R_{⊥⊥}^{A}}\right)}^{2}+{\left(\frac{\tau_{n1}\left(\theta \right)}{R_{⊥∥}}\right)}^{2}+{\left(\frac{p_{⊥\psi }^{c}}{R_{⊥\psi }^{A}}\sigma_{n}\left(\theta \right)\right)}^{2}}\;\;\;+\frac{p_{⊥\psi }^{c}}{R_{⊥\psi }^{A}}\sigma_{n}\left(\theta \right)$$

(13) $$\begin{array}{c} R_{⊥}^{At}=R_{⊥}^{t}\;;\;R_{⊥∥}^{A}=R_{⊥∥}\;;\;R_{⊥⊥}^{A}=\frac{R_{⊥}^{c}}{2\left(1+p_{⊥⊥}^{c}\right)}\\ \frac{p_{⊥\psi }^{t,c}}{R_{⊥\psi }^{A}}=\frac{p_{⊥⊥}^{t,c}}{R_{⊥⊥}^{A}}\cos^{2}\psi +\frac{p_{⊥∥}^{t,c}}{R_{⊥∥}^{A}}\sin^{2}\psi \\ \cos^{2}\psi =\frac{\tau_{nt}^{2}}{\tau_{nt}^{2}+\tau_{n1}^{2}}\;;\;\sin^{2}\psi =\frac{\tau_{n1}^{2}}{\tau_{nt}^{2}+\tau_{n1}^{2}}\\ p_{⊥⊥}^{c}=\frac{1}{2\cdot \cos^{2}\theta }-1\;;\;\frac{p_{⊥⊥}^{c}}{R_{⊥⊥}^{A}}=\frac{p_{⊥∥}^{c}}{R_{⊥∥}}\\ \cos\theta_{fp}=\sqrt{\frac{{\left(\frac{\tau_{21}}{\sigma_{2}}\right)}^{2}\cdot {\left(\frac{R_{⊥⊥}^{A}}{R_{⊥∥}}\right)}^{2}+1}{2\cdot \left(1+p_{⊥⊥}^{c}\right)}} \end{array}$$

where $R_{⊥}^{At}$ and $R_{⊥⊥}^{A}$ are the tensile and shear strengths in the transverse direction of the fiber; $R_{⊥∥}^{A}$ is the shear strengths in the longitudinal direction of the fiber; $R_{⊥}^{c}$ is the compression strength perpendicular to the fiber direction; $p_{⊥⊥}^{t,c}$, $p_{⊥∥}^{t,c}$ are the inclination parameters; and $\theta_{fp}$ is the fracture angle of the failure plane. The strength values of the Puck failure criterion are listed in Table 6.

The failure occurred when stress risk factor $f_{E,FF}$ or $f_{E,IFF}$ was greater than 1. Stiffness degradation of the failure elements was performed according to different stiffness-degradation models. Stiffness-degradation criteria [33] are depicted in Table 7.

## 4. Results and Discussion

**Figure 10:**
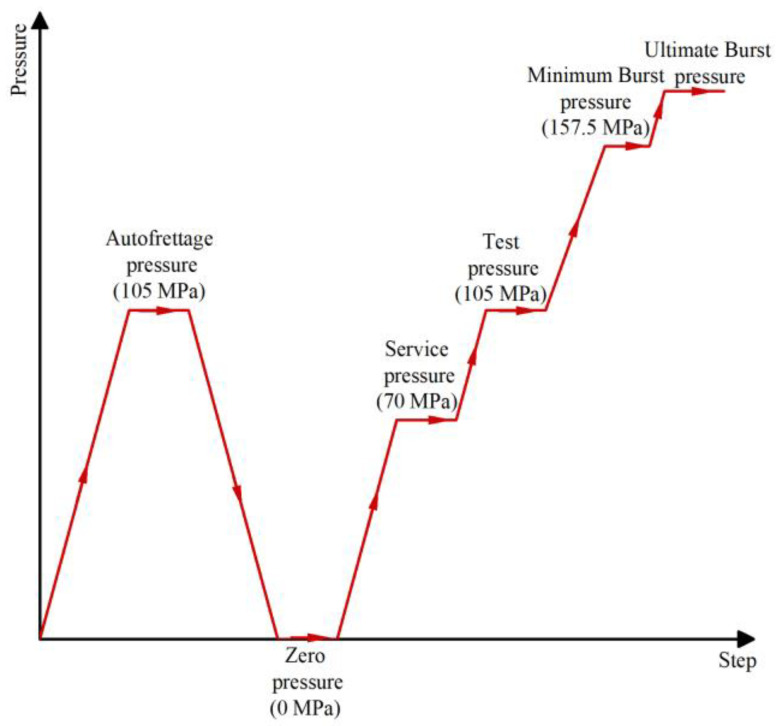
Loading history for FEM of hydrogen storage composite vessels.

The loading history for vessels, as shown in Figure 10, was used in this paper. The working pressure of hydrogen storage vessels was 70 MPa. The safety factor was set as 2.25. The minimum burst pressure was 157.5 MPa. If the hydrogen storage vessels were not structurally damaged at 157.5 MPa, the internal pressure was increased until burst to obtain the ultimate burst pressure of the hydrogen storage vessels.

### 4.1. Load-Bearing Capacity and Damage Behavior

**Figure 11:**
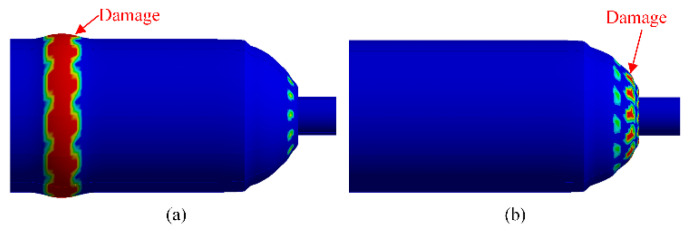
Comparison of numerical results of two burst modes: (**a**) safe mode, (**b**) unsafe mode.

The explosion simulations were carried out to predict the burst pressures of hydrogen storage vessels. Two burst modes, as shown in Figure 11, may occur; i.e., the safe burst mode and unsafe burst mode. The safe burst mode was attributed to fiber breakage in hoop fibers. The failure appeared in the cylindrical section while the port of the dome section entered the vessel. During this burst, the axial displacement was decreased rapidly after its value reached the maximum displacement point. The unsafe burst mode was attributed to fiber breakage in helical fibers. The failure appeared in the dome section, and the port was ejected at high speed.

**Figure 12:**
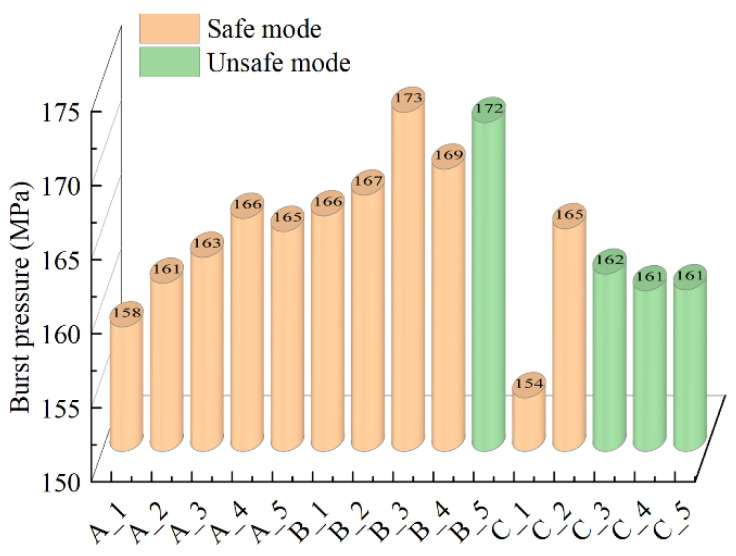
Numerical results of the burst pressure and the burst mode.

Figure 12 shows the burst pressure and the burst mode of the vessels of different layer-up sequences. The results showed that the reaming process reduced fiber thickness near polar openings. The reaming process improved the stress distribution of composite layers, which increased the burst pressure of vessels. The reaming process increased the helical winding angle. The larger the helical winding angle was, the more hoop-bearing capacity could be increased. The schemes A_1 and B_3 showed that the burst pressure of the hydrogen storage vessel with reaming was increased by 9.15%.

The polar openings of the dome section bore not only the internal pressure of its area but also the internal pressure of the port area. A greater bearing capacity was required in composite layers near polar openings than in the other part. The scheme B_5 showed that the load-bearing capacity of the cylindrical section was improved by the reaming process. However, the strength of composite layers at polar openings was reduced by the reaming process, which made the vessel change from the safe mode to the unsafe mode. The load-bearing capacity of the vessel could be improved by using a reasonable reaming process under the premise that the vessel was in safe mode. From the results of groups A and B, it was suggested that the number of reaming layers was less than half of the design number of helical layers.

**Figure 13:**
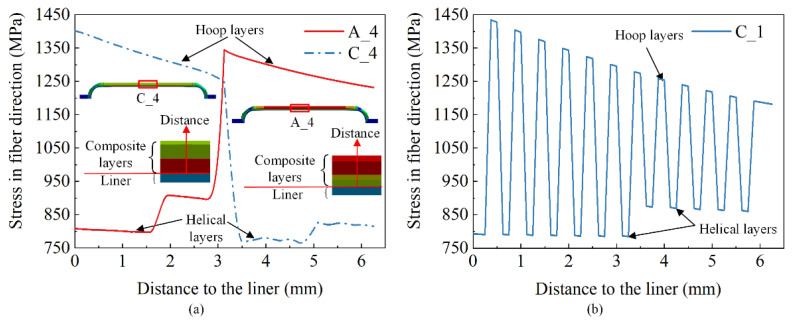
Comparison of numerical results of the stress in fiber direction at the cylindrical section: (**a**) Schemes A_4 and C_4, (**b**) Scheme C_1.

Figure 13 shows fiber stress of schemes A_4, C_4, and C_1 along the thickness direction of composite layers at the cylindrical section at the test pressure. The helical layers stress was less than the hoop layers stress in the cylindrical section under internal pressure. The fiber strength of helical layers was not fully utilized. The hoop fibers were broken first when the burst appeared in the cylindrical section. The outer portion of composite layers, which consisted of hoop layers, could increase the average stress of the helical layers of the cylindrical section by 5.92% and reduce the average stress of hoop layers by 3.65%.

The ultimate load-bearing capacity of vessels was determined by the maximum stress in the fiber direction. The smaller the stress was, the safer the vessel was. The bearing capacity of vessels could be increased by raising the fiber strength utilization factor of helical layers. The increase in bearing capacity could increase the burst pressure of the hydrogen storage vessels. A gradient change of stress along the thickness direction was caused by the adoption of hoop and helical alternated model, and the stress decreased as the number of layers increased. A large stress difference between the inner and the outer of hoop layers prevented outer hoop layers from completing enough work under the burst pressure, which was not conducive to the utilization of fiber strength. The safest and the highest burst pressure was reached by changing the layer-up sequence as follows: the hoop and helical layers were separated, the winding angles of helical layers were changed from small to large, and the inner portion of the composite layers consisted entirely of helical layers.

**Figure 14:**
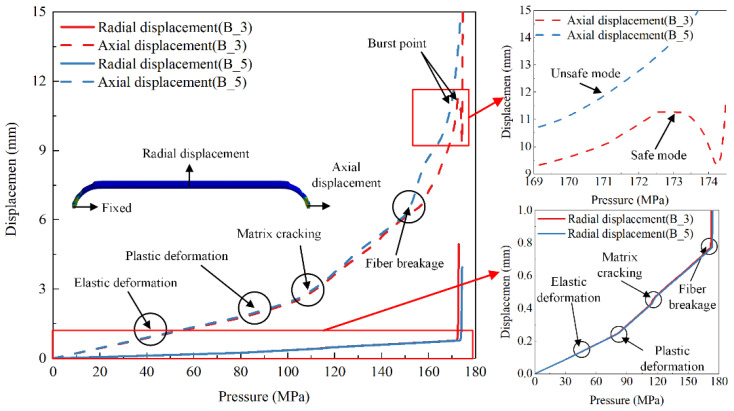
Comparison of numerical results of load-displacement curve between schemes B_3 and B_5.

Figure 14 shows the axial displacement (axial displacement at polar openings of the dome) and the radial displacement (radial expansion of the cylindrical section) curves of schemes B_3 and B_5 under internal pressure loading. The burst mode of vessels could be predicted by the curves. The axial displacement curve of the scheme B_3 presented the burst point, which showed that it was under the safe burst mode. The axial displacement of scheme B_5 continued increasing, which showed it was under the unsafe burst mode.

The radial and axial displacement curves of the two schemes showed both an obvious linear and nonlinear phase before the vessels were structurally damaged. The initial linearity represented that the material was in the elastic phase. The nonlinear state of curves was caused by elastoplastic deformation of the aluminum alloy inner liner and the damage of composite layers. There were four regions before the vessel was structurally damaged, as shown in the curves of scheme B_3. The first linear curve before 85 MPa denoted the initial elastic behavior of the aluminum liner. The second nonlinear curve before 113 MPa denoted the plastic deformation of the aluminum liner. The third nonlinear curve before 158 MPa indicated resin matrix damage, but the composite vessel still had a great load-bearing capacity. Finally, a sharply rising curve showed that fiber damage occurred at about 158 MPa, and the vessel burst at about 172.9 MPa. The fracture behavior of the fiber determined the load-bearing capacity of vessels, but the matrix damage had little effect on the ultimate load-bearing capacity of vessels.

### 4.2. Damage-Evolution Laws

**Figure 15:**
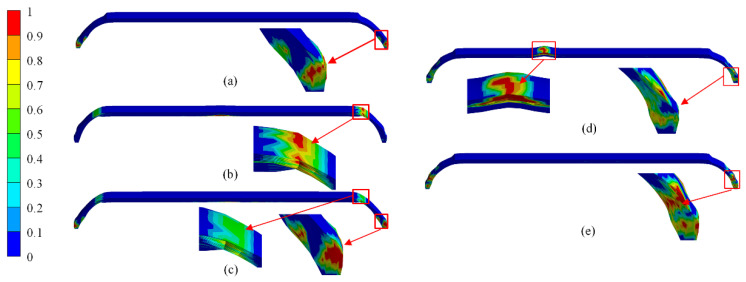
Comparison of numerical results of fiber damage at the burst pressure: (**a**) Scheme C_3, (**b**) Scheme C_4, (**c**) Scheme C_5, (**d**) Scheme B_3, and (**e**) Scheme B_5.

As shown in Figure 15a–c, the fiber damaged under the burst pressure of schemes C_3, C_4, and C_5, which were all under the unsafe burst mode. The damage of scheme C_3 appeared at polar openings of the dome section. The damage of scheme C_4 appeared at the connection between cylindrical and dome section, and the damage of scheme C_5 appeared at polar openings of the dome section and the connection between cylindrical and dome section. The fiber overhead occurred when helical angles were changed from large to small, or the inner portion of composite layers consisted entirely of hoop layers. The fiber overhead caused stress concentration and reduced the load-bearing capacity of the dome section, and the unsafe burst mode occurred.

The fiber damage of schemes B_3 and B_5 under the burst pressure is shown in Figure 15d,e. The fiber of hoop layers in the cylindrical section of scheme B_3 was completely damaged under the burst pressure, causing penetration damage. The fiber of helical layers at the dome section was partially damaged without causing structural damage. However, the fiber damage at the dome section of scheme B_5 accumulated continuously to form a penetration trend. The fiber of the cylindrical section was not damaged when structural damage occurred in the hydrogen storage vessel.

**Figure 16:**
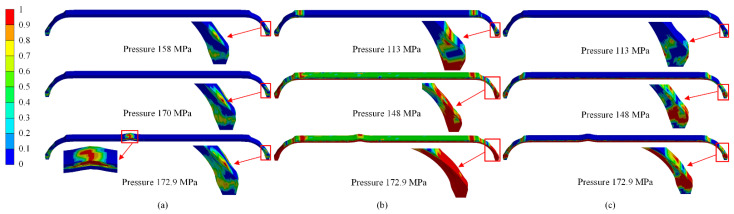
Numerical results of progressive damage of the composite layers in the composite hydrogen storage vessels: (**a**) fiber tensile, (**b**) matrix tensile, and (**c**) matrix compression.

Figure 16 shows the damage evolution of fiber and matrix in the scheme B_3. The fiber was mainly subject to tensile stress, and almost no compression failure occurred when the hydrogen storage vessel was under working pressure. Therefore, only fiber tensile damage and matrix tensile and compression damage were shown. A matrix failure occurred at polar openings and the connection between cylindrical and dome sections when internal pressure increased to 113 MPa. The low strength of resin caused the matrix damage. As internal pressure increased, matrix damage developed from the inner to the outside of composite layers. Although the matrix in the dome section had been severely damaged, fiber was not yet broken. The hydrogen storage vessel had no structural damage. The fiber failure occurred at polar openings of the dome section with internal pressure increasing to 158 MPa. This was not enough to cause structural damage to the hydrogen storage vessel because the fiber damage area was small. The fiber tensile failure extended to other areas as the internal pressure increased. Finally, the hoop fibers of the cylindrical section were damaged with an internal pressure increase to 172.9 MPa. The hoop fibers’ damage resulted in stiffness degradation, and the damage rapidly expanded from the inside to the outside of the composite layers. The damage formed a penetrating area and caused structural damage to the hydrogen storage vessel. Research on the damage evolution behavior of the hydrogen storage vessel showed that the ultimate load-bearing capacity of hydrogen storage vessels largely depended on the fiber strength. The matrix had little effect on the burst pressure of hydrogen storage vessels.

### 4.3. Stress Distribution of the Aluminum Liner

**Figure 17:**
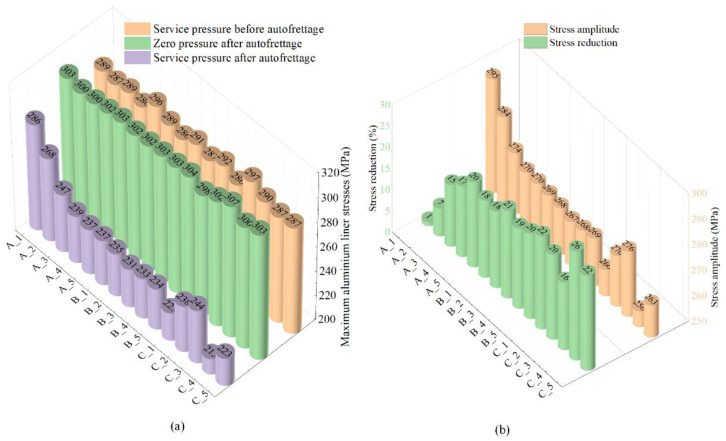
Numerical results of the maximum stress of the aluminum liner: (**a**) under different pressure; (**b**) stress reduction and stress amplitude.

Figure 17 shows the maximum stress of the aluminum liner under different pressures. Each column represents the maximum stress of the aluminum liner. The stress reduction was defined as the change of the maximum stress of the aluminum liner before and after autofrettage at the working pressure. The stress amplitude was defined as half of the difference between the maximum stress of the aluminum liner under the working pressure and the zero pressure.

The maximum stress and the stress amplitudes under the working pressure after autofrettage showed a downward trend as the reaming times and layers increased. The stress distribution of the aluminum liner under the working pressure was improved after the reaming process. The stress reduction in the aluminum liner under the working pressure improved the fatigue performance of vessels. Scheme B_3 showed that the maximum stress of the aluminum liner was reduced by 20.59% after the reaming process, and the stress amplitude was reduced by 9.28%. As the reaming times and layers increased further, the strength of the dome section decreased, and the burst mode changed from the safe mode to the unsafe, but the maximum stress and the stress amplitudes just decreased slightly.

Group C showed that the stress distribution of the aluminum liner was affected by the layer-up sequence. The maximum stress and the stress amplitude of scheme C_1, which adopted hoop and helical layers alternated model, were improved the most, except for the schemes of the unsafe burst mode. The fatigue performance of hydrogen storage vessels was improved by adopting the alternated model. However, the burst pressure of scheme C_1 was only 153.62 MPa. Scheme A_4, which had the same layers and winding angles as scheme C_1, adopted the model with hoop and helical layers separated. The burst pressure of scheme A_4 was 165.73 MPa and was increased by 7.88%. The results showed that the burst pressure of hydrogen storage vessels was improved by adopting the model with hoop and helical layers separated.

**Figure 18:**
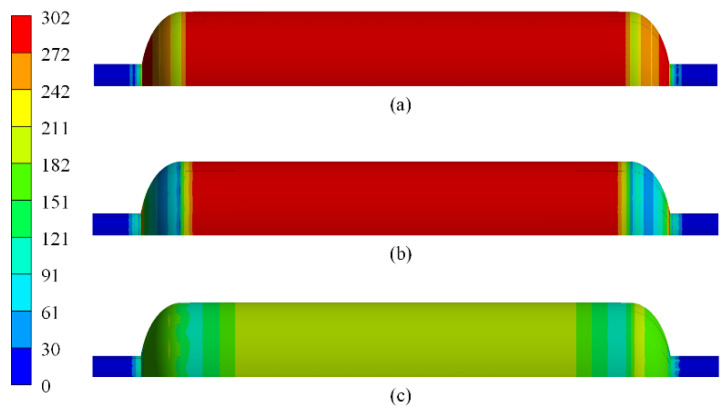
Numerical results of loading history of the aluminum liner at (**a**) service pressure before autofrettage, (**b**) zero pressure after autofrettage, and (**c**) service pressure after autofrettage.

Figure 18 shows the stress distribution of the aluminum liner of scheme B_3. The maximum stress of the aluminum liner under working pressure was 291.41 MPa before autofrettage, which exceeded the yield strength of the material. Residual tensile stress was produced in composite layers after autofrettage. The residual compressive stress of the aluminum liner was caused by the restriction of the composite layers. The maximum stress of the aluminum liner under working pressure was reduced to 237.97 MPa, and the fatigue performance of the vessel was improved after autofrettage.

### 4.4. Comparison with the Cubic Spline Function Method

**Figure 19:**
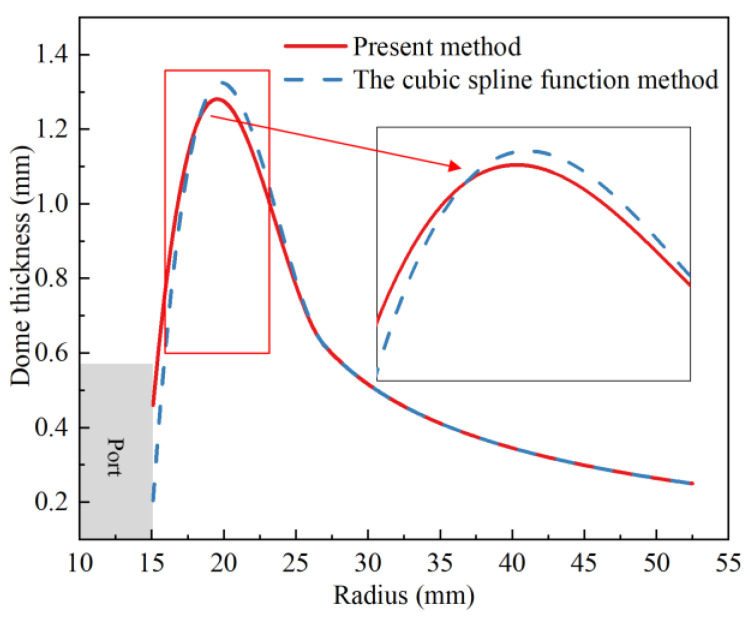
Comparison of analytical results of dome thickness between two methods.

The comprehensive performance of the scheme B_3 was the best. For comparison, the cubic spline function method was used to analyze the hydrogen storage vessel of scheme B_3. Figure 19 shows the distribution curves of dome thickness predicted by the present method and the cubic spline function method. The cubic spline function method underestimated dome thickness at polar openings, and a sharp thickness peak occurred at about one bandwidth. The curve predicted by the present method was smoother and avoided a sharp thickness peak at about one bandwidth. The present method provided more reasonable and accurate results.

**Figure 20:**
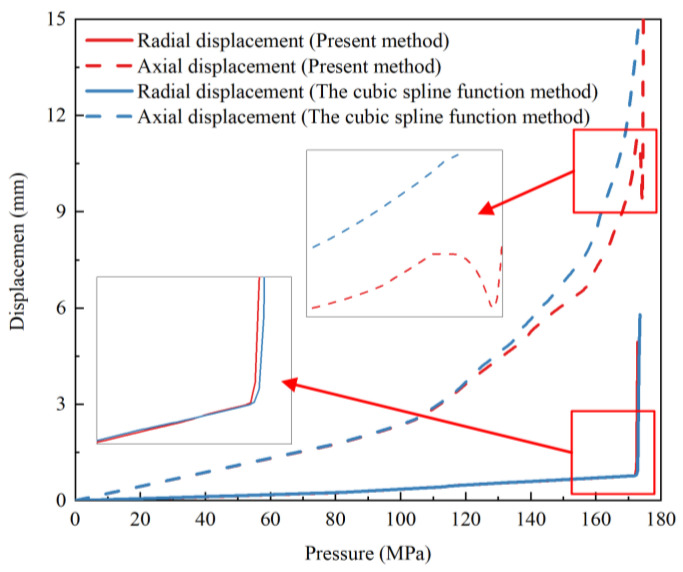
Comparison of numerical results of load-displacement curves between two methods.

A finite-element model of the vessel was established by using the dome thickness predicted by the cubic spline function method. The burst pressure obtained by the finite-element analysis was 173.3 MPa, with little difference from the 172.9 MPa predicted by the present method. The radial displacement curves of the two methods were similar, as shown in Figure 20. The axial displacement curves of the two methods had no obvious difference before 113 MPa but separated after 113 MPa. The results showed that the pressure of matrix damage was 113 MPa. The axial displacement curve of the cubic spline function method increased more obviously than the curve of the present method. The axial displacement curve of the cubic spline function method did not present a burst point, which showed that the burst mode of the vessel predicted by the cubic spline function method was unsafe.

**Figure 21:**
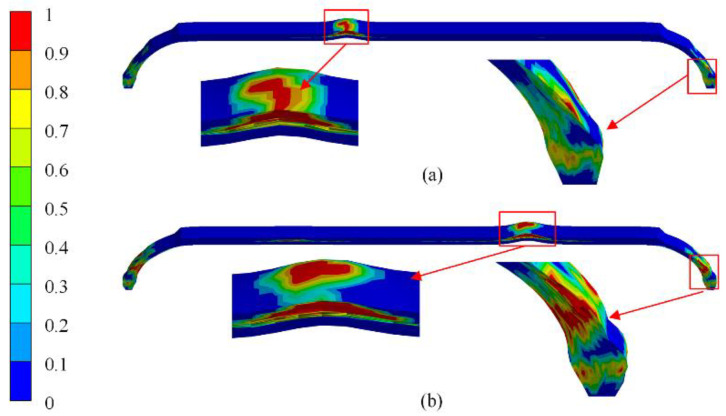
Comparison of numerical result of the fiber damage at the burst pressure: (**a**) present method, (**b**) the cubic spline function method.

As shown in Figure 21, the fibers in the dome section were partially damaged under the burst pressure from the results of the present method, but not enough to cause structural damage. The cubic spline function method showed that the fiber damage in the dome section eventually formed a penetration area, resulting in structural damage. The cubic spline function method underestimated the actual thickness of polar openings, leading to a lower strength of composite layers. The finite-element results predicted by the present method were closer to the actual results.

## 5. Conclusions

In the present research, a method for predicting the thickness of the dome section was proposed, which involved the effect of fiber slippage and the redistribution of fiber tows. This prediction method was used to perform progressive damage analysis on composite hydrogen storage vessels. The analysis results were compared with the results of the cubic spline function method. The following conclusions can be deduced:

(1) The maximum relative error of the predicted thickness was 4.19%, the mean absolute percentage error was 2.04%, and the root-mean-square error was 0.48 mm. The results verified that the predicted thickness of the present method was in good agreement with the actual measured thickness.
(2) The results showed that the reaming process increased the burst pressure of the hydrogen storage vessel by 9.15%. The maximum stress of the aluminum liner under the working pressure was reduced by 19.15% after autofrettage, and the stress amplitude was reduced by 9.28%.
(3) The stress concentration in the composite layers caused by fiber overhead was improved by changing the layer-up sequence as follows: the hoop and helical layers were separated, the winding angles of helical layers were changed from small to large, and the inner portion of the composite layers consisted of helical layers. The burst pressure of the vessel was increased by 7.88% compared with the traditional design sequence.
(4) The burst pressures predicted by the two methods had little difference. However, the cubic spline function method underestimated the actual thickness of polar openings. The burst mode of the vessel predicted by the cubic spline function method was unsafe. The analysis results of the present method approached the actual case.

## Figures and Tables

**Table 1:** The parameters of three composite vessels.

Vessel’s Type	Dome Length *h* (mm)	Fiber Band *b* (mm)	Thickness of Fiber Tows *t_p_* (mm)	Polar Radius *r*_0_ (mm)	Radius of Cylinder Section *R* (mm)
a	80	5.39	0.765	9.5	132
b	213	5.39	0.765	60	132
c	213	12.5	0.48	110	372.5

**Table 2:** Relative error between the analytical results and the measurement results.

		The Present Method		The Double Formula Method		The Cubic Spline Function Method	
*r* (mm)	Actual Thickness (mm)	Predicted Value (mm)	Relative Error (%)	Predicted Value (mm)	Relative Error (%)	Predicted Value (mm)	Relative Error (%)
9.50	8.50	8.25	−2.94	0	-	5.31	-
11.20	22.00	21.12	−4.00	20.69	−5.95	21.77	−1.05
11.60	23.50	22.96	−2.29	22.67	−3.53	24.05	2.34
12.50	26.00	25.69	−1.12	26.27	1.04	27.34	5.15
13.50	27.30	26.77	−1.94	29.42	7.77	28.42	4.10
14.50	26.60	26.19	−1.57	31.98	20.23	27.28	2.56
15.40	24.80	24.63	−0.69	24.23	−2.30	25.10	1.21
16.30	21.50	22.40	4.19	19.73	−8.23	22.13	2.93
17.50	18.70	18.93	1.23	16.54	−11.55	18.00	−3.74
18.56	15.80	15.96	1.01	14.69	−7.03	15.00	−5.06
19.50	13.80	13.81	0.07	13.45	−2.54	13.38	−3.04
20.30	13.00	12.63	−2.85	12.59	−3.15	12.45	−4.23

**Table 3:** Material properties of aluminum alloy 6061-T6.

Property	E (GPa)	μ	σ_s_ (MPa)	σ_b_ (MPa)	δ (%)	ρ (kg/m^3^)
6061-T6	74.12 GPa	0.28	340	281	11.57	2700

**Table 4:** Material properties of the T700/epoxy composite.

Property	E_11_ * (GPa)	E_22_ * (GPa)	E_33_ * (GPa)	G_12_ (MPa)	G_23_ (MPa)	G_13_ (MPa)	μ_12_	μ_23_	μ_13_
T700/epoxy composite	134	7.42	7.42	3710	4790	3710	0.28	0.3	0.28

* Direction 1—fiber direction, Direction 2—transverse direction, Direction 3—normal to layer direction.

**Table 5:** Material properties of the T700/epoxy composite.

Property	X_T_ (MPa)	X_C_ (MPa)	Y_T_ (MPa)	Y_C_ (MPa)	S_12_ (MPa)	S_23_ (MPa)	S_13_ (MPa)	ρ (kg/m^3^)
T700/epoxy composite	2300	1250	74	180	50	90	50	1680

Direction 1—fiber direction, Direction 2—transverse direction, Direction 3—normal to layer direction.

**Table 6:** Parameters of Puck criterion.

Parameter	$R_{∥}^{t}\;(\mathrm{MPa})$	$R_{∥}^{c}\;(\mathrm{MPa})$	$E_{∥}\;(\mathrm{GPa})$	$E_{∥f}\;(\mathrm{GPa})$	$\nu_{⊥∥}$	$\nu_{⊥∥f}\;$
Valve	2300	1250	230	134	0.28	0.2
$R_{⊥}^{At}$ (MPa)	$R_{⊥∥}^{A}$ (MPa)	$R_{⊥}^{c}$ (MPa)	$p_{⊥⊥}^{t,c}$	$p_{⊥∥}^{t}$	$p_{⊥∥}^{c}$	
74	50	180	0.3	0.3	0.35	

**Table 7:** Stiffness-degradation criteria of composite materials.

Failure Modes	Stiffness-Degradation Criteria
Fiber tensile failure	*E*_11_ = 0.07*E*_11_, *E*_22_ = *E*_33_ = 0.07*E*_22_, *υ*_12_ = *υ*_13_ = 0.07*υ*_12_, *υ*_23_ = 0.07*υ*_23_, *G*_12_ = *G*_13_ = 0.07*G*_12_, *G*_23_ = 0.07*G*_23_
Fiber compression failure	*E*_11_ = 0.14*E*_11_, *E*_22_ = *E*_33_ = 0.14*E*_22_, *υ*_12_ = *υ*_13_ = 0.14*υ*_12_, *υ*_23_ = 0.14*υ*_23_, *G*_12_ = *G*_13_ = 0.14*G*_12_, *G*_23_ = 0.14*G*_23_
Matrix tensile failure	*E*_22_ = 0.2*E*_22_, *G*_12_ = 0.2*G*_12_, *G*_23_ = 0.2*G*_23_
Matrix compression failure	*E*_22_ = 0.4*E*_22_, *G*_12_ = 0.4*G*_12_, *G*_23_ = 0.4*G*_23_

## Data Availability

The data presented in this study are available on request from the corresponding author.
